# Molecular phylogeny implies exclusive, repetitive, convergent establishment of cyanobacterial symbiosis in didemnid ascidians (Tunicata: Ascidiacea: Didemnidae)

**DOI:** 10.1186/s40851-026-00272-9

**Published:** 2026-08-29

**Authors:** Mamiko Hirose, Euichi Hirose

**Affiliations:** 1 Department of Zoology, National Museum of Nature and Science, Tsukuba, Ibaraki, 305-0005 Japan; 2 Department of Chemistry, Biology and Marine Science, Faculty of Science, University of the Ryukyus, Nishihara, Okinawa, 903-2213 Japan

**Keywords:** COI, Multiple origins, Obligate symbiosis, Oxygenic photosymbiosis, *Prochloron*, *Synechocystis*, Vertical transmission

## Abstract

Despite the great diversity of the phylum Chordata, lifelong obligate symbiosis with cyanobacteria is only known in certain species belonging to four genera of didemnid ascidians: *Didemnum*, *Diplosoma*, *Lissoclinum*, and *Trididemnum*. This photosymbiosis in didemnid ascidians exhibits diversity in photosymbiont taxa, symbiont location in the host ascidian, and modes of vertical transmission of symbionts. While the family Didemnidae comprises over 600 colonial species, approximately 30 symbiotic species are known from tropical waters, alongside many non-symbiotic congeners. This study aimed to elucidate the evolutionary history of this unique photosymbiosis in the family Didemnidae by constructing molecular phylogenetic trees based on the partial sequences of two regions in the mitochondrial cytochrome *c* oxidase subunit I gene (*COI*) and mapping the character states of photosymbiosis in each ascidian species harboring obligate photosymbionts onto the trees. The molecular phylogenetic trees generally supported the monophyly of *Didemnum*, *Diplosoma*, and *Lissoclinum*, but not that of *Trididemnum*. In contrast, the phylogenetic tree did not support the monophyly of the following character states of obligate photosymbiosis in ascidians: photosymbiont taxa (*Prochloron*, *Synechocystis*, and filamentous species), photosymbiont location (tunic, tunic cells, and peribranchial/common cloacal cavities), and modes of vertical transmission of photosymbionts in which the symbionts are attached to the larval trunk, collected by the rastrum, or transferred by tunic cells. These results suggest that obligate photosymbiosis with similar character states may have been exclusively established multiple times within the family Didemnidae. Because the modes of vertical transmission are closely associated with symbiont location, similar modes of vertical transmission may have evolved multiple times under similar colony structures and reproductive modes.

**Supplementary Information:**

The online version contains supplementary material available at https://doi.org/10.1186/s40851-026-00272-9.

## Background

Oxygenic photosymbiosis has been found in various metazoan taxa, such as Porifera, Cnidaria, Xenacoelomorpha, Mollusca, and Chordata. Some host animals establish obligate symbiosis with photosymbionts (e.g., cyanobacteria and microalgae), whereas the association is facultative in others [1]. Typically, obligate photosymbiosis is recognized by the presence of photosymbionts in every conspecific individual or colony. Among chordates, obligate, lifelong photosymbiosis has been exclusively recognized in some ascidian species belonging to the family Didemnidae, which comprises colonial species only [2, 3]. In other chordate taxa, although embryos of the spotted salamander associate with green algae, this association is facultative and limited to embryonic stages [4]. The family Didemnidae is the largest family in the subphylum Tunicata (=Urochordata), comprising nine genera and over 600 species [5]. The monophyly of this family is widely accepted based on synapomorphies, such as pyloric budding [6, 7], and is also supported by 18S rRNA phylogenies [8, 9]. To date, nine genera have been distinguished in Didemnidae based on several morphological character states, including the presence or absence of an atrial siphon for each zooid, presence or absence of calcareous spicules, shape of testis lobe(s), shape of the vas deferens, and number of rows of stigma [10, 11]. Approximately 30 species of four didemnid genera (*Didemnum, Diplosoma*, *Lissoclinum*, and *Trididemnum*) have established obligate symbiosis with cyanobacteria, such as *Prochloron* and *Synechocystis* [11–16]. Host ascidians are distributed in tropical and subtropical shallow waters [2, 11, 14, 17]. The occurrence of ascidian–cyanobacterial symbiosis in warm waters is likely caused by the vulnerability of cyanobacterial symbionts to low temperatures [18].

Why is obligate photosymbiosis exclusively found in didemnid ascidians among the various chordate taxa, whereas only facultative associations have been reported in other taxa? In obligate photosymbiosis of didemnid ascidians, photosymbionts are always transferred to the progeny (ascidian larvae) examined so far, but vertical transmission of photosymbionts has never been reported in species with facultative associations with cyanobacteria [3, 19–29]. Therefore, vertical transmission is likely a necessary condition for the establishment of an obligate relationship between the host ascidian and cyanobacteria. However, it is unclear why vertical transmission was exclusively established in some didemnids but not in other ascidian species, regardless of whether they were colonial or solitary. To better understand the exclusive occurrence of obligate photosymbiosis in this unique chordate taxon, it is important to recognize the diversity of symbiosis systems in didemnid ascidians and its evolutionary history in the didemnid phylogeny.

The diversity of photosymbiosis in didemnids can be recognized by three characteristics [3]: (1) cyanobacterial species, (2) location of the photosymbionts in the host ascidian colony, and (3) mode of vertical transmission. As summarized in Table 1, multiple types of symbiosis systems have been found in each host genus, whereas the intracellular type is only known in *Lissoclinum punctatum* Kott, 1977 [30]. Obligate photosymbiosis is thought to have arisen independently at least once in each photosymbiotic didemnid genus. The restriction of larval adaptations for algal transmission to obligately photosymbiotic species has been considered evidence for this hypothesis [20, 22]. If so, did the similar mode of vertical transmission recognized across different genera evolve in a convergent manner? To discuss this assumption, it is necessary to verify the monophyly of each genus as well as the monophyly of this family.

**Table 1:** Diversity of obligate photosymbiosis in didemnid ascidians

Host genus	Symbionts	Location of symbionts	Mode of vertical transmission	Case of host species
*Didemnum*	*Prochloron*	Cloacal cavity	Attached on larval trunk	*D. molle* [22, 24]
		Tunic	Unknown	*D. viride* [22]
*Trididemnum*	*Prochloron*	Cloacal cavity	Attached on larval trunk	*T. cyclops* [20]
		Tunic	Carried by tunic cells	*T. miniatum* [20, 25]
	*Synechocystis*	Tunic	Carried by tunic cells	*T. nubilum* [28]
	Multiple species			*T. clinides* [28]
*Lissoclinum*	*Prochloron*	Cloacal cavity	Attached on larval trunk	*L. patella* [20]
		Cloacal cavity & tunic cells		*L. punctatum* [27, 30]
		Tunic	Carried by tunic cells	*L. midui* [29]
*Diplosoma*	*Prochloron*	Cloacal cavity	Collected with rastrum	*D. simile* [23]

Molecular phylogenetic studies of ascidians of the family Didemnidae, based on the sequences of the 18S rRNA gene and/or partial sequences of the mitochondrial cytochrome *c* oxidase subunit I gene (*COI*), have not denied the monophyly of each didemnid genus, except *Trididemnum* [8, 31, 32], and are therefore consistent with the aforementioned hypothesis that obligate photosymbiosis has multiple origins within Didemnidae. However, to consider the evolutionary history of obligate symbiosis based on various modes of symbiosis, the datasets are insufficient, as species with different modes of symbiosis are not adequately covered. Although the didemnid molecular phylogeny analyzed by Oliveira et al. [32] dealt with the largest number of genera and species, they did not mention photosymbiosis in didemnid lineages. Although we have provided partial *COI* sequences as DNA barcodes for some obligately photosymbiotic didemnids in Japan and Taiwan [8, 33], the region of these *COI* sequences (Yokobori region) is different from the universal region (Folmer region) used in many other studies, such as Oliveira et al. [32]. Therefore, our sequence data for DNA barcodes cannot be used with the data provided in other studies, although a dataset is required to cover many taxa and samples of symbiotic and non-symbiotic species for better phylogenetic analysis.

This study aimed to provide the first comprehensive evolutionary framework for photosymbiosis in Didemnidae by integrating the diversity of symbiotic modes (i.e., symbiont identity, symbiont location, and vertical transmission mode) with a molecular phylogeny based on the broadest taxon sampling of both photosymbiotic and non-photosymbiotic didemnid species to date. Although previous molecular phylogenetic studies have included photosymbiotic didemnids, none has explicitly examined the evolution of photosymbiosis in relation to the diversity of symbiotic modes. We sequenced the universal region of *COI* for 54 specimens representing 18 photosymbiotic didemnid species and the Yokobori region for 18 specimens representing 13 species. By combining these data with the dataset of Oliveira et al. [32] and publicly available sequence data, we reconstructed a phylogeny based on the universal region of *COI*, yielding a tree that included 48 species from six didemnid genera. We also reconstructed a phylogeny using the combined universal and Yokobori regions, resulting in a tree that included 23 species from five genera. Finally, by mapping photosymbiotic character states onto these phylogenies, we discuss the hypothesis that cyanobacterial symbioses have evolved repeatedly and convergently within Didemnidae.

## Materials and methods

### Animals

Didemnid colonies were collected by snorkeling in the shallow subtidal zone between 2005 and 2018. The collection sites for each specimen are listed in Table 2. The authors collected all specimens and identified the species. The colonies were subsequently preserved in 99% (v/v) ethanol at −30 °C for DNA analysis. Some parts of colonies were also fixed in 10% formalin-seawater after anesthesia with menthol and 0.37 M MgCl_2_ and dissected for species identification with reference to [11, 14, 16]. The formalin-fixed specimens were subsequently transferred to 70% (v/v) ethanol and donated to the Ryukyu University Museum Fujukan (RUMF) as voucher specimens. Some samples lacked voucher specimens because all colonies were consumed during DNA extraction and dissection.

**Table 2:** List of didemnid samples sequenced in this study

Genus	Species	Collection Site Sampling date	Specimen-ID/Abbreviation	Voucher ID	Acc. No.	Region	Symbiont*
*Didemnum*							
	*Did. molle*	Seragaki, Okinawajima Is., Okinawa, Japan 2007/4/16	A022		LC905408	Folmer	P
	*Did. molle*	Bise, Okinawajima Is., Okinawa, Japan 2007/4/16	A027		LC905409	Folmer	P
	*Did. molle*	Singapore 2013/5/25	A187	RUMF:ZU-00021	LC905410	Folmer + Yokobori	P
*Diplosoma*							
	*Dip. aggregatum*	Muiga, Miyakojima Is., Okinawa, Japan 2008/8/2	A097	RUMF:ZU-00022	LC905411	Folmer + Yokobori	P
	*Dip. aggregatum*	Imgya, Miyakojima Is., Okinawa, Japan 2008/8/3	A102	RUMF:ZU-00023	LC905412	Folmer	P
	*Dip. aggregatum*	Dabaisha, Green Is., Taiwan 2017/5/18	A263	RUMF:ZU-00024	LC905413	Folmer	P
	*Dip. gumavirens*	Hirakubo, Ishigakijima Is. Okinawa, Japan 2007/10/26	A012	RUMF:ZU-00025	LC905414	Folmer + Yokobori	P
	*Dip. gumavirens*	Shinrihama Beach, Kumejima Is., Okinawa, Japan 2007/8/28	A053	RUMF:ZU-00026	LC905415	Folmer	P
	*Dip. gumavirens*	Penghu, Taiwan 2014/8/28	A211	RUMF:ZU-00027	LC905416	Folmer	P
	*Dip. gumavirens*	Pari Is., Indonesia 2013/11/28	A196		LC905417	Folmer + Yokobori	P
	*Dip. gumavirens*	Pari Is., Indonesia 2013/11/29	A198	MZB:Asc. 00006	LC905418	Folmer	P
	*Dip. gumavirens*	Dabaisha, Green Is., Taiwan 2017/5/18	A264	RUMF:ZU-00028	LC905419	Folmer	P
	*Dip. gumavirens*	Gueiwan, Green Is., Taiwan 2017/5/19	A267	RUMF:ZU-00029	LC905420	Folmer	P
	*Dip. ooru*	Shiraho, Ishigakijima Is., Okinawa, Japan 2007/10/25	A008	RUMF:ZU-00030	LC905421	Folmer	P
	*Dip. ooru*	Nanwan, Kenting, Taiwan 2011/3/23	A126	RUMF:ZU-00031	LC905422	Folmer	P
	*Dip. ooru*	Honba, Minami-Daito, Okinawa, Japan 2013/3/13	A180	RUMF:ZU-00032	LC905423	Folmer + Yokobori	P
	*Dip. ooru*	Kaigunbou, Minami-Daito, Okinawa, Japan 2013/3/13	A184	RUMF:ZU-00033	LC905424 LC905425	Folmer Yokobori	P
	*Dip. ooru*	Dabaisha, Green Is., Taiwan 2017/5/18	A262	RUMF:ZU-00034	LC905426	Folmer	P
	*Dip. simile*	Ishijirou Beach, Hahajima, Tokyo, Japan 2012/5/14	A168	RUMF:ZU-00036	LC905427	Folmer + Yokobori	P
	*Dip. simile*	Bise, Okinawajima Is., Okinawa, Japan 2016/9/2	A222	RUMF:ZU-00037	LC905428	Folmer + Yokobori	P
	*Dip. simile*	Bise, Okinawajima Is., Okinawa, Japan 2016/9/2	A223	RUMF:ZU-00038	LC905429	Folmer	P
	*Dip. simile*	Bise, Okinawajima Is., Okinawa, Japan 2016/9/2	A224		LC905430	Folmer	P
	*Dip. simileguwa*	Nanwan, Kenting, Taiwan 2011/3/23	A125	NMNS: 6650–001	LC905431	Folmer	P
	*Dip. simileguwa*	Pari Is., Indonesia 2013/11/28	A197	MZB:Asc. 00008	LC905432	Folmer	P
	*Dip. simileguwa*	Ooura, Okinawajima Is., Okinawa, Japan 2014/1/14	A209	RUMF:ZU-00039	LC905433	Folmer + Yokobori	P
	*Dip. variostigmatum*	Hirakubo, Ishigakijima Is. Okinawa, Japan 2007/10/26	A014	NSMT:PC 1109	LC905434	Folmer + Yokobori	P
	*Dip. variostigmatum*	Teniya, Okinawajima Is., Okinawa, Japan 2008/8/15	A052	RUMF:ZU-00040	LC905435	Folmer	P
	*Dip. variostigmatum*	Bise, Okinawajima Is., Okinawa, Japan 2016/9/2	A219	RUMF:ZU-00041	LC905436	Folmer + Yokobori	P
	*Dip. virens*	Teniya, Okinawajima Is., Okinawa, Japan 2007/9/13	A062	RUMF:ZU-00042	LC905437	Folmer + Yokobori	P
	*Dip. virens*	Uganzaki, Ishigakijima Is., Okinawa, Japan 2011/9/13	A148	RUMF:ZU-00043	LC905438	Folmer	P
	*Dip. watanabei*	Shimama, Tanegashima Is., Kagoshima, Japan 2006/8/10	Yoko150	NUM:Az 0645	LC905439	Folmer + Yokobori	P
	*Dip. watanabei*	Ngamahama, Kurimajima Is., Okinawa, Japan 2008/12/10	A075	RUMF:ZU-00044	LC905440	Folmer	P
	*Dip. watanabei*	Yobuko-hama, Tonakijima Is., Okinawa, Japan 2012/3/21	A161	RUMF:ZU-00045	LC905441	Folmer + Yokobori	P
*Leptoclinides*							
	*Lep. echinatus*	Nabeta, Shimoda, Shizuoka, Japan 2005/5/27	Yoko134		LC905442	Folmer + Yokobori	N
	*Lep. madara*	Okinoshima, Tateyama, Chiba, Japan 2014/5/14	A210	RUMF:ZU-00046	LC905443 LC905444	Folmer Yokobori	N
*Lissoclinum*							
	*Lis. bistratum*	Bise, Okinawajima Is., Okinawa, Japan 2016/9/2	A227	RUMF:ZU-00047	LC905445 LC905446	Folmer Yokobori	P
	*Lis. midui*	Gongguan, Green Is., Taiwan 2017/9/7	A275	RUMF:ZU-00048	LC905447	Folmer	P
	*Lis. midui*	Shinrihama Beach, Kumejima Is., Okinawa, Japan 2018/5/30	A280	RUMF:ZU-00049	LC905448	Folmer	P
	*Lis. midui*	Shinrihama Beach, Kumejima Is., Okinawa, Japan 2018/5/30	A281	RUMF:ZU-00050	LC905449	Folmer	P
	*Lis. midui*	Shinrihama Beach, Kumejima Is., Okinawa, Japan 2018/5/30	A282	RUMF:ZU-00051	LC905450	Folmer	P
	*Lis. punctatum*	Odo, Okinawajima Is., Okinawa, Japan 2016/12/10	A260	RUMF:ZU-00052	LC905451	Folmer + Yokobori	P
	*Lis. timorense*	Bise, Okinawajima Is., Okinawa, Japan 2016/9/2	A228	RUMF:ZU-00056	LC905452 LC905453	Folmer Yokobori	P
	*Lis. timorense*	Imgya, Miyakojima Is., Okinawa, Japan 2008/8/3	A103	RUMF:ZU-00053	LC905454	Folmer	P
	*Lis. timorense*	Uganzaki, Ishigakijima Is., Okinawa, Japan 2011/9/13	A149	RUMF:ZU-00054	LC905455	Folmer	P
	*Lis. timorense*	Pari Is., Indonesia 2013/11/30	A199	RUMF:ZU-00055	LC905456	Folmer	P
*Trididemnum*							
	*T. clinides*	Zanpa, Okinawajima Is., Okinawa, Japan 2008/3/21	A083		LC905457 LC905458	Folmer Yokobori	M
	*T. clinides*	Odo, Okinawajima Is., Okinawa, Japan 2008/3/22	A084	RUMF:ZU-00057	LC905459	Folmer	M
	*T. cyclops*	Hirakubo, Ishigakijima Is. Okinawa, Japan 2007/10/26	A016	RUMF:ZU-00058	LC905460	Folmer	P
	*T. cyclops*	Ishijirou Beach, Hahajima, Tokyo, Japan 2012/5/14	A169	RUMF:ZU-00059	LC905461	Folmer	P
	*T. miniatum*	Bise, Okinawajima Is., Okinawa, Japan 2017/8/21	A271	RUMF:ZU-00060	LC905462	Folmer	P
	*T. miniatum*	Bise, Okinawajima Is., Okinawa, Japan 2017/8/21	A272	RUMF:ZU-00061	LC905463	Folmer	P
	*T. miniatum*	Bise, Okinawajima Is., Okinawa, Japan 2017/8/21	A273	RUMF:ZU-00062	LC905464	Folmer	P
	*T. miniatum*	Bise, Okinawajima Is., Okinawa, Japan 2017/8/21	A274	RUMF:ZU-00063	LC905465	Folmer	P
	*T. nubilum*	Ara Beach, Kumejima Is., Okinawa, Japan 2008/6/5	A088		LC905466	Folmer	S
	*T. nubilum*	Bise, Okinawajima Is., Okinawa, Japan 2017/8/21	A270		LC905467	Folmer	S
	*T. paracyclops*	Agari-hama, Tonakijima Is., Okinawa, Japan 2012/3/22	A162	RUMF:ZU-00065	LC905468	Folmer	P

* P, *Prochloron*; S, *Synechocystis*; M, multiple species; N, non-symbiotic

The occurrence of cyanobacterial symbionts was checked in each sample, and generic identification was performed based on the color and morphology of the symbiotic algae. For the species in Oliveira et al. [32], the presence of photosymbionts and their genera were based on the literature on the host species [11–15].

### DNA extraction, amplification, and sequencing

DNA was extracted from tissue samples preserved in ethanol at −30 °C. Genomic DNA was extracted from approximately 30–40 zooids using a DNeasy Tissue Kit (Qiagen K.K., Tokyo, Japan) following the manufacturer’s protocol. A segment of the “Yokobori region” in the mitochondrial COI gene was amplified using the primers UroCox1-244F and UroCox1-387 R, following the method described by Hirose et al. [34]. Following subcloning, five clones were randomly selected for sequencing.

**Fig. 1:**
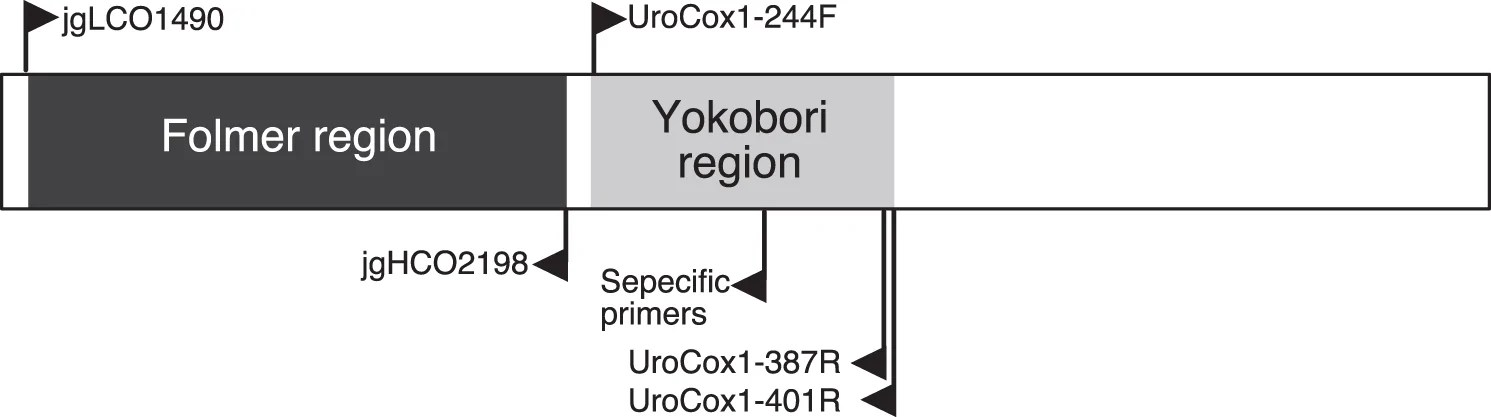
Schematic diagram of COI gene showing the position used for PCR amplification and sequence of Folmer and Yokobori regions. Arrowheads denote positions of primers in this study (Table 3)

PCR with marine invertebrate universal primers jgLCO1490 and jgHCO2198 [35] was performed to amplify and obtain the ‘universal region’ (=Folmer region) sequence under the following conditions: 94 °C for 5 min, followed by 35 cycles at 94 °C for 30 s, 50 °C for 30 s, and 72 °C for 1 min 30 s, and 72 °C for 7 min for final extension. Nested PCR was subsequently employed when this approach failed. The primers UroCox-401 R (specific for ascidian species (Yokobori unpublished)) and jgLCO1490 were used for the first round of amplification. One microliter (1 µL) of the PCR product was used for the second round of amplification in the nested PCR protocol using the primer pairs jgLCO1490 and specific primers (Table 3, Fig. 1). The first PCR conditions were 94 °C for 5 min, followed by 35 cycles at 94 °C for 30 s, 48 °C for 30 s, 72 °C for 1 min 30 s, and 72 °C for 7 min for final extension. The second PCR conditions were 94 °C for 5 min, followed by 35 cycles at 94 °C for 30 s, 50 °C for 30 s, 72 °C for 1 min 30 s, and 72 °C for 7 min for final extension. PCR products were treated with ExoSAP-IT (Affymetrix, California, USA) prior to sequencing reactions. Sequencing reactions were performed by Operon Biotechnology (Tokyo, Japan). The sequences determined in this study were deposited in the DDBJ/EMBL/GenBank databases under the accession numbers LC905408 - LC905468.

**Table 3:** PCR primers used in this study

Primer	Sequence (5’–3’)	Specificity	Citation
jgLCO1490	TITCIACIAAYCAYAARGAYATTGG	Invertebrate	[35]
jgHCO2198	TAIACYTCIGGRTGICCRAARAAYCA	Invertebrate	[35]
UroCox1-244F	CATTTWTTTTGATTWTTTRGWCATCCNGA	Ascidian	[34]
UroCox1-387 R	GCWCYTATWSWWAAWACATAATGAAARTG	Ascidian	[34]
UroCox1-401 R	SCHACRACRTARTAWGTRTCRTGHA	Ascidian	Yokobori unpublished
YokoD196R	GCWGTAAARTAAGCYCGWGTRTC	*Didemnum, Diplosoma*	This study
LmYokoR122	AAACCAATGCTACATATAGCTCAAA	*Lissoclinum midui*	This study
LmYokoR228	TCTCGTAGGAATAGCGATAA	*Lissoclinum midui*	This study
TmYokoR260	GCAGCAGTAAAATAAGCTCGAGTA	*Trididemnum miniatum*	This study
TmYokoR425	AAACCGGAATTAGCTAAAACAA	*Trididemnum miniatum*	This study

### Molecular analyses

An analysis of the universal region (universal tree) was performed based on 119 OTUs, which included 56 sequences obtained in this study (Table 2) and 63 sequences retrieved from databases (Supplementary Table S1). Among the species listed in Table 2 and Table S1, *Prochloron* or other cyanobacteria occasionally colonize the colony surface of *Didemnum multispiral*e Kott, 2001, *Lissoclinum verrilli* (Van Name, 1909), and *Trididemnum pigmentatum* Kott, 2001, and chlorophytes are occasionally embedded in the surface tunic of *Didemnum psammatodes* (Sluiter, 1895) [11, 36]. In these species, some conspecific colonies often harbor no photosymbionts, indicating that the association is facultative rather than obligate symbiosis.

An analysis of the combined sequences from the universal and Yokobori regions (combined tree) was conducted using 42 OUTs, comprising 31 sequences obtained in this study and 11 sequences retrieved from the database (Table S1). In some species, since neither the universal nor Yokobori region sequences could be obtained from the same batch (clonal group) of genomic DNA, sequences from different batches collected at the same locations were combined and used for phylogenetic analysis. For species *T. miniatum* and *L. midui*, concatenated sequences were constructed only after confirming sequence consistency among overlapping regions and/or correspondence with an available complete mitochondrial genome. For *T. miniatum* from Okinawajima Island, nucleotide sequences were concatenated only between two specimens (ID A272 (LC905463) and A044 (AB813827)) collected from the same locality. The resulting concatenated sequence was identical to the corresponding region of the previously published complete mitochondrial genome. For *L. midui*, nucleotide sequences were concatenated only between specimens collected from the same locality. Specifically, sequences from Green Island were concatenated from specimens ID A275 (LC905447) and A191 (AB899783), whereas those from Kumejima Island were concatenated from specimens A280 (LC905448) and A054 (AB602780). No concatenation was performed between specimens from different localities.

First, DNA nucleotide sequences were translated into amino acid sequences using the ascidian mitochondrial genetic code (NCBI translation table 13). Using these amino acid sequences, we performed sequence alignment and identified the insertion/deletion regions. Based on this amino acid alignment, initial DNA sequence alignments were generated using MUSCLE [37], followed by visual inspection and manual editing of the alignments. MEGA version 11.0 [38] was used to select an appropriate nucleotide substitution model. For the datasets including all codon positions, the GTR + I + G model was selected as the best-fit model for both the universal and combined trees based on the Bayesian Information Criterion (BIC) and the Akaike Information Criterion (AIC). Maximum likelihood (ML) analysis was performed, and bootstrap support (1,000 replicates) was estimated using RAxML-NG version 1.2.2 [39]. Bootstrap support for the resulting phylogenetic tree was estimated using the neighbor-joining (NJ) and maximum parsimony (MP) methods (1,000 replicates) in MEGA version 11.0.

Intra- and interspecific genetic distances (K2P-corrected) were calculated using MEGA version 11.0. The resulting distances were plotted to visualize barcoding gaps using ggplot2 version 4.0.0.

## Results

### DNA barcoding gap and species discrimination

**Fig. 2:**
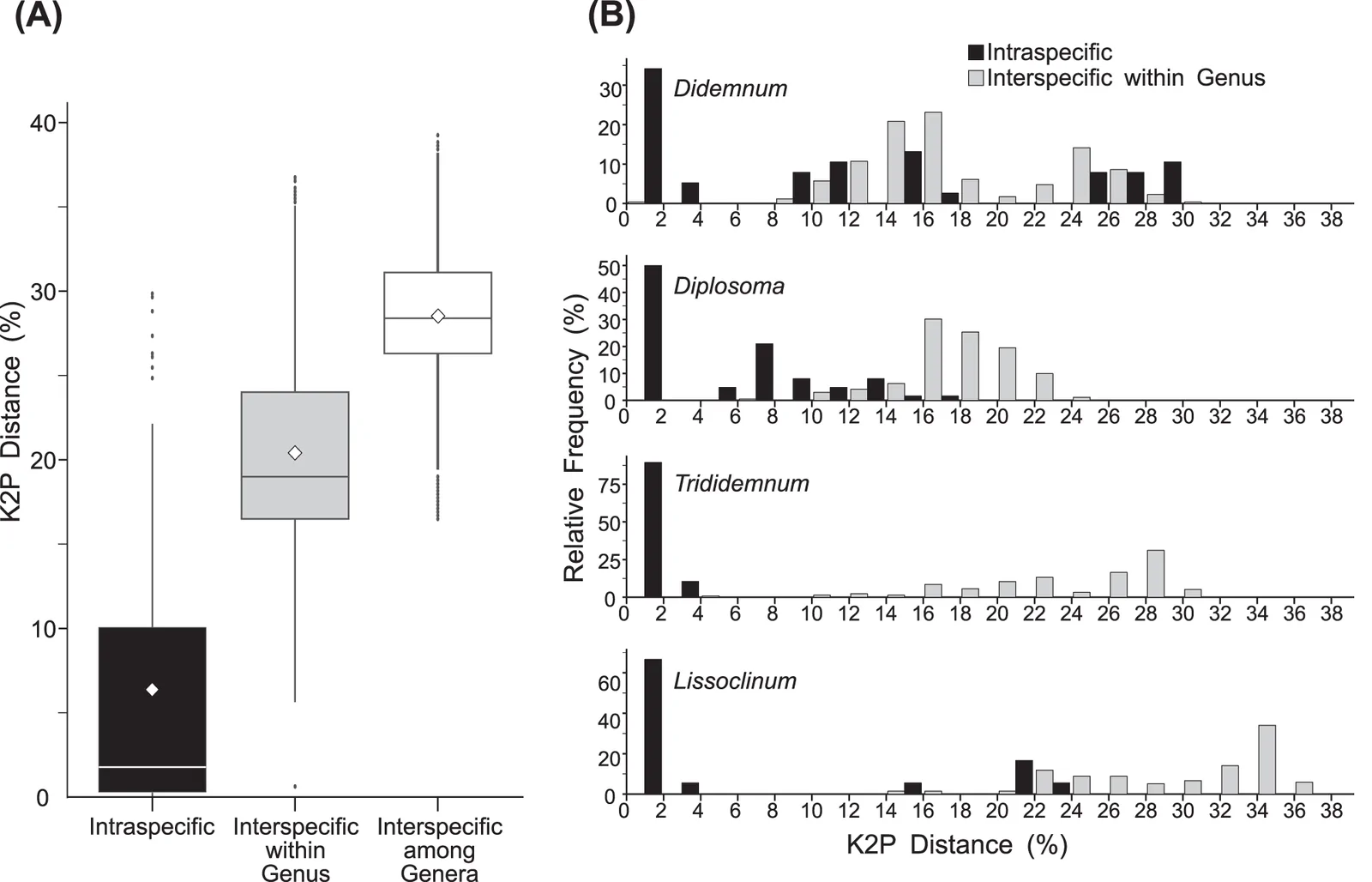
DNA barcoding gaps of interspecific and intraspecific genetic distance in the universal region of *COI* (480 bp), based on the K2P model. (**A**) Boxplot of intraspecific, interspecific within genus, and interspecific among genera distance. Each box from top to bottom represents the maximum, upper quartile, median, lower quartile, and minimum of the interspecific distance. The white rhombus indicates the mean intraspecific genetic distance. (**B**) Histogram of the calculated intraspecific and interspecific within-genus distances for each analyzed Didemnidae specimen

Before using COI sequences for phylogenetic inference, we evaluated whether they reliably discriminated morphologically identified species by examining intra- and interspecific genetic distances. To evaluate the utility of COI sequences as a DNA barcode for species discrimination, we compared intra- and interspecific genetic distances and examined the presence of a barcoding gap. Genetic distances were calculated using the K2P model based on 116 sequences from 27 didemnid species included in the phylogenetic analysis of the universal region (480 bp) (Table 4). The mean values for intraspecific variation, interspecific variation within each genus, and interspecific variation among genera were 6.3 ± 8.2% (average ± standard deviation, *n* = 139), 20.4 ± 5.9% (*n* = 1478), and 28.5 ± 3.5% (*n* = 5169), respectively. Significant differences were observed among all three categories (Kruskal–Wallis test followed by Dunn’s multiple comparison test, *p* < 0.01; Fig. 2A).

**Table 4:** Inter-specific divergence between congeneric species and intra-specific variation of didemnid *COI* universal region (481 bp)

	All	*Didemnum*	*Diplosoma*	*Leptoclinides*	*Lissoclinum*	*Polysyncraton*	*Trididemnum*
No. of species	27	14	10	2	9	2	10
No. of OTU	116	34	37	3	18	3	21
Intra species							
No. of combination	139	38	62	1	18	1	19
Average (%)	6.3	11.7	4.8	19.4	6.2	0.0	0.5
SD (%)	8.2	10.9	4.7	NA	9.2	NA	0.8
Max (%)	29.9	29.9	17.4	19.4	22.1	0.0	2.3
Min (%)	0.0	0.0	0.0	19.4	0.0	0.0	0.0
Inter species with genus							
No. of combination	1478	523	604	2	135	2	212
Average (%)	20.4	18.5	18.4	20.7	30.5	26.9	24.5
SD (%)	5.9	5.2	2.9	0.9	5.4	0	5.3
Max (%)	36.7	31.3	24.4	21.3	36.7	26.9	31.7
Min (%)	0.6	0.6	6.7	20.0	15.9	26.9	5.6

NA: Not available. SD: Standard deviation

In the analysis of the genus *Didemnum*, 34 sequences from 14 species were examined. Despite the large intraspecific variation and overlap in the frequency distributions of K2P distances between intra- and interspecific variation (Fig. 2B), a significant difference was still detected between the two categories (Mann–Whitney U test, *p* < 0.01). In Table 4, within the genera *Diplosoma*, *Lissoclinum*, and *Trididemnum*, intraspecific variation ranged from 0.5 to 6.2%. For each genus with three or more species, the ratio of the interspecific genetic distance within the genus (genus-level average, %) to the intraspecific average (%) ranged from approximately 3.8-fold to 49-fold. In these three genera, the K2P distances showed that intraspecific variation was significantly lower than the interspecific variation in congeners. The frequency distributions of K2P distances for intraspecific and interspecific variation overlapped across all genera, and no clear barcoding gaps were observed.

### Universal tree

**Fig. 3:**
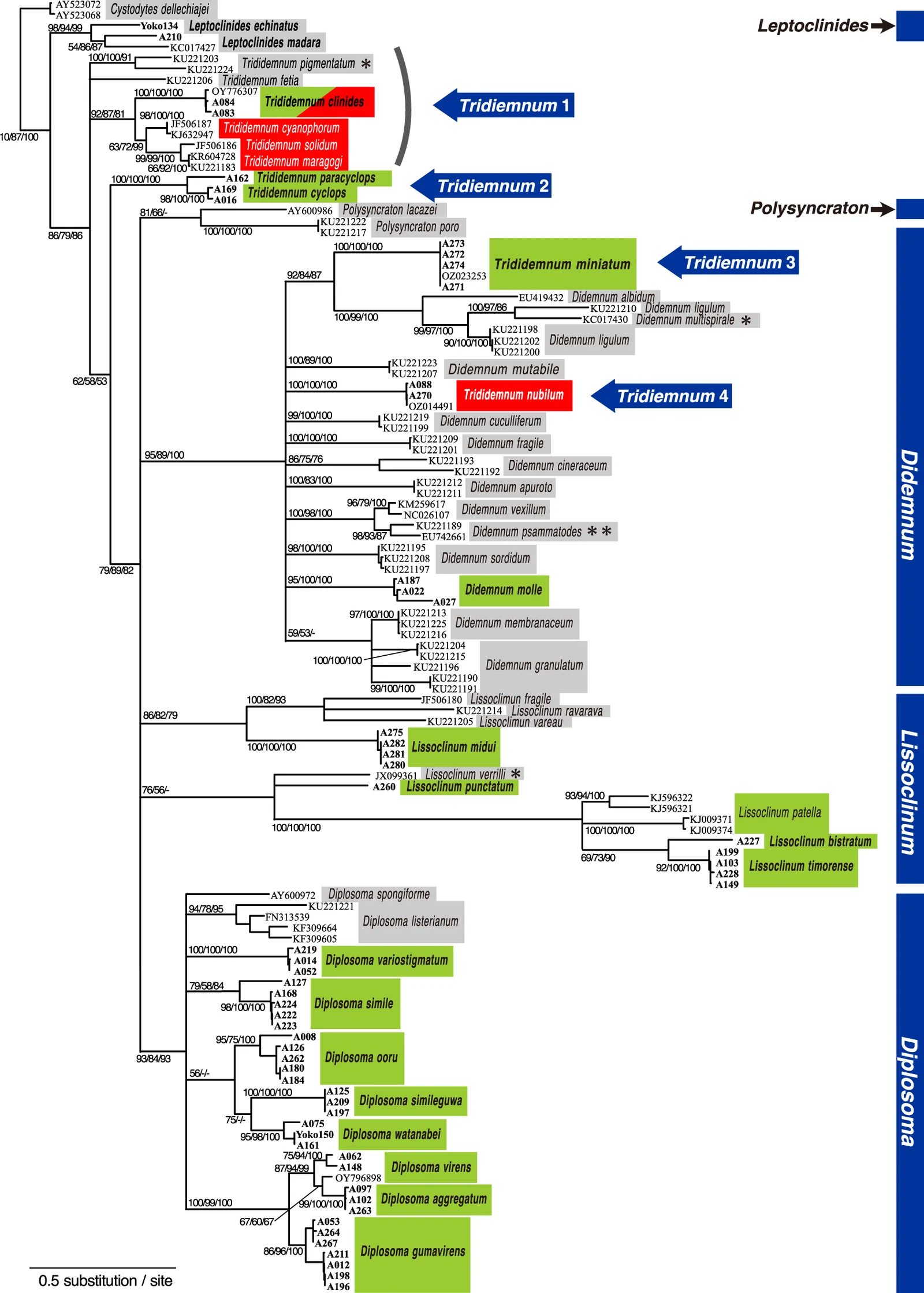
Universal tree: maximum likelihood (ML) tree of didemnid species based on universal region of cytochrome *c* oxidase subunit I (*COI*) gene sequences. The GTR + I+ G model was used for the analysis. Bootstrap probabilities larger than 50% for the ML, maximum-parsimony (MP), and neighbor-joining (NJ) analyses are noted. The color of the background of each species name indicates the presence of obligate cyanobacterial symbiont: green for *Prochloron*, red for *Synechocystis*, green/red for multiple species, and gray for the absence of obligate cyanobacterial symbionts. *cyanobacterial cells occasionally colonize the colony surface. **chlorophytes are occasionally embedded in the surface tunic

A total of 119 operational taxonomic units (OTU) comprising 480 base pairs were used for phylogenetic analysis, with *Cystodytes dellechiajei* (Della Valle, 1877) designated as the outgroup (Fig. 3). All insertions/deletions were deleted using MEGA software. All species form well-supported monophyletic clades. Sequences from *Didemnum* and *Diplosoma* each form well-supported monophyletic clades (*Didemnum* ML = 95%, MP = 89%, NJ = 100%; *Diplosoma* ML = 93%, MP = 84%, NJ = 93%, respectively). Sequences from *Lissoclinum* cluster into two clades with moderate support (one was ML = 86%, MP = 82%, NJ = 79%; the other was ML = 76%, MP = 56%, respectively); the monophyly of the genus was not supported. Photosymbiotic and non-photosymbiotic species were often found in the same monophyletic clade. The monophyly of the genus *Trididemnum* was not supported. In the present study, *Trididemnum* was divided into four groups. *Trididemnum* 1 comprises six species, including non-symbiotic species, species symbiotic with *Synechocystis*, and species symbiotic with multiple cyanophytes. *Trididemnum* 2 consists of *T. cyclops* Michaelsen, 1921 and *T. paracyclops* Kott, 1980, both of which are symbiotic with *Prochloron*. *Trididemnum* 3 consists of *T. miniatum*, which is symbiotic with *Prochloron*, and *Trididemnum* 4 consists of *T. nubilum* Kott, 1980, which is symbiotic with *Synechocystis*; both belong to the *Didemnum* clade.

### Combined tree

**Fig. 4:**
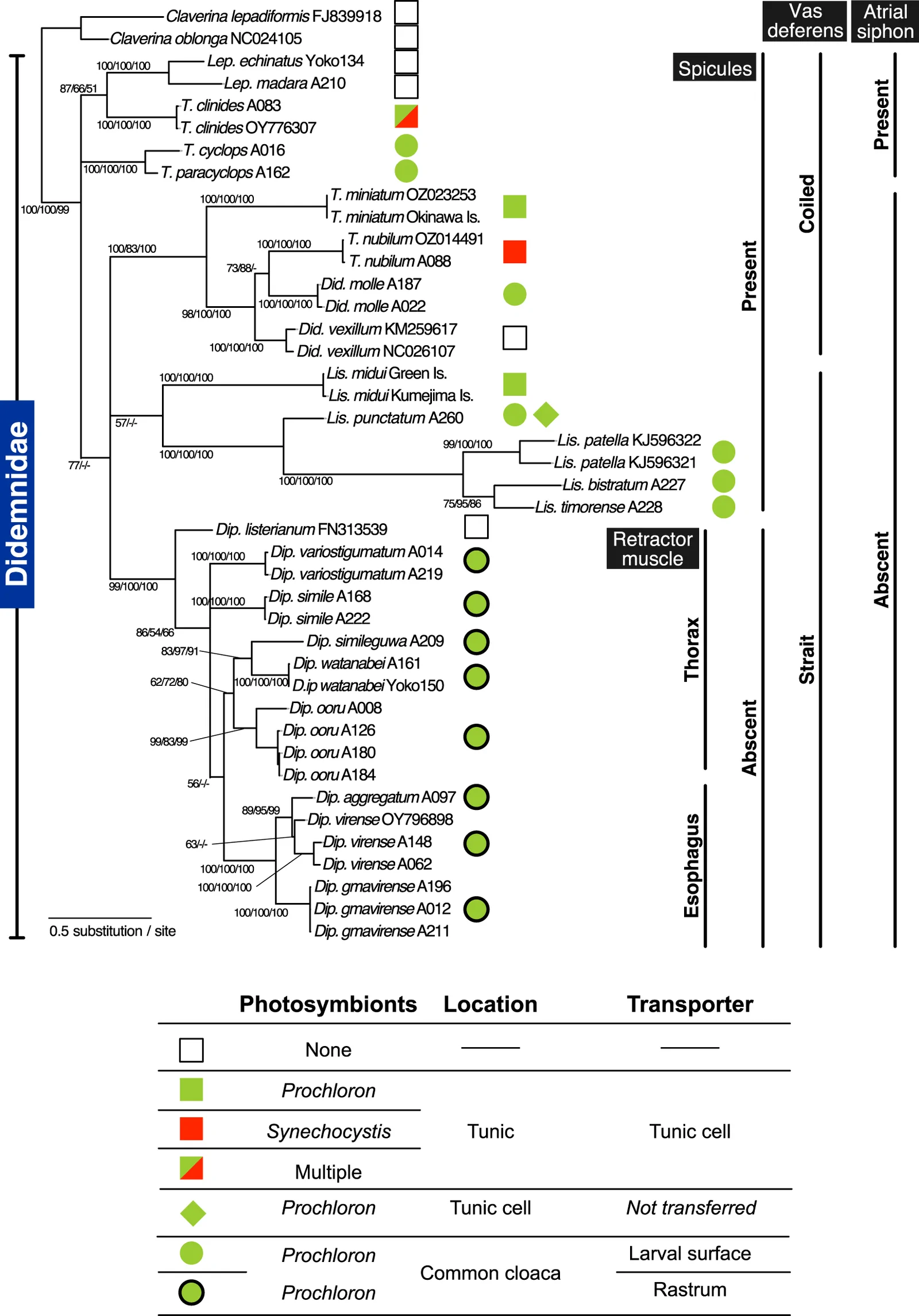
Combined tree: maximum likelihood (ML) tree of didemnid species based on the combined sequence of universal and Yokobori region of cytochrome *c* oxidase subunit I (*COI*) gene. The GTR + I+ G model was used for the analysis. Bootstrap probabilities larger than 50% for the ML, maximum-parsimony (MP), and neighbor-joining (NJ) analyses are noted. The symbols next to the species names indicate taxon, location, and mode of vertical transmission of the cyanobacterial symbionts (see the explanatory table). The bars on the right-hand side of the phylogenetic tree denote morphological characters.

A total of 42 OTU comprising 918 base pairs were used for phylogenetic analysis, with *Clavelina lepadiformis* (Müller, 1776) and *C. oblonga* Herdman, 1880 designated as outgroups (Fig. 4). The monophyly of the genus *Diplosoma* was highly supported (ML = 99%, MP = 100%, NJ = 100%), whereas the monophyly of the genus *Lissoclinum* was poorly supported (ML = 57%, MP < 50%, NJ < 50%). The monophyly of the genus *Trididemnum* was not supported, and as in the universal tree, it was divided into four groups. In Fig. 4, the symbols following the species names indicate photosymbiont taxa, their location in the host colony, and the mode of vertical transmission. Regardless of the genus (excluding *Diplosoma*), cyanobacterial cells located in the cloacal cavity were directly attached to the larval trunk, and those embedded in the tunic were transferred to the embryo’s tunic by the tunic cells [3]. In the monophyletic clade of *Diplosoma,* no species harbors photosymbionts in its tunic, and cyanobacterial cells in the cloacal cavity are directly attached to the rastrum of the pre-hatching larva. Some morphological character states were mapped onto the combined tree (Fig. 4); the presence or absence of an atrial siphon, form of the vas deferens, presence or absence of spicules, and liberating position of the retractor muscle do not contradict the inferred phylogenetic relationships.

## Discussion

### Utility of mtCOI sequences for species identification and phylogenetic inference

Before discussing the evolutionary history inferred from the phylogenetic tree, it is important to evaluate whether the *COI* sequences used in this study provide reliable species-level identification. Obtaining *COI* barcode sequences from didemnid ascidians has often been challenging because of their highly divergent mitochondrial genomes. The DNA barcoding system, developed by Hebert et al. [40] and based primarily on partial *COI* sequences, enables molecular identification of species. In birds, insects, and fish, interspecific variation typically exceeds intraspecific variation, resulting in a distinct DNA barcode gap [41]. In contrast, in two groups of amphibians (frogs and mantellid salamanders), intraspecific variation overlaps with interspecific variation when only model-based genetic distances (K2P) are considered [42]. However, phylogenetic analyses support the monophyly of each species, demonstrating the utility of these markers as DNA barcodes. Although some genera in this study did not exhibit clear barcoding gaps, the phylogenetic relationships inferred from partial *COI* sequences were nevertheless strongly supported. Therefore, although *COI* barcodes alone cannot resolve all taxonomic questions, they provide sufficiently reliable species identification to support phylogenetic analyses of photosymbiotic didemnids.

### Phylogeny of didemnid genera and taxonomical character states

The family Didemnidae is often characterized by pyloric (esophageal) budding, stellate to globular calcareous spicules embedded in the tunic, and three or four rows of stigmata [43]. Because this mode of budding is exclusively unique to didemnid species, the monophyly of this family has rarely been questioned. Furthermore, molecular phylogeny based on 18S rDNA or partial *COI* sequences ([8, 9, 32] and this study) and cladistic analyses [7] support the monophyly of Didemnidae.

At the genus level, the present and preceding molecular phylogenetic trees [32, 44] agree well with the current morphological classification systems but also have some inconsistencies. Phylogenetic analyses, including six of the nine didemnid genera *Didemnum*, *Diplosoma*, *Leptoclinides*, *Lissoclinum*, *Polysyncraton*, and *Trididemnum*, supported the monophyly of each genus in trees based on 18S rDNA [32]. However, the monophyly of *Trididemnum* was not supported due to polytomies in the tree based on the partial *COI* sequences of the universal region [32] and Yokobori region [44]. In the present study, the tree based on the universal region of *COI* sequences (universal tree) supported the monophyly of *Diplosoma*, *Leptoclinides*, and *Polysyncraton*, whereas *Lissoclinum* formed two clades, and *Trididemnum* was divided into four groups (*Trididemnum* 1–4). *Didemnum* formed a single clade with *Trididemnum* 3 (*T. miniatum*) and 4 (*T. nubilum*). In the tree based on sequences combining the universal and Yokobori regions (combined tree), *Diplosoma* and *Leptoclinides* formed monophyletic clades, whereas the monophyly of *Lissoclinum* was poorly supported by bootstrap values (Fig. 4). *Trididemnum* remained polyphyletic in this tree, and *Didemnum* formed a monophyletic clade with *T. miniatum* and *T. nubilum*.

Among didemnid genera, *Trididemnum* is defined by three rows of stigmata on the branchial sac, whereas all other didemnid species have four rows of stigmata. The polyphyly of *Trididemnum* species indicates that the reduction of stigma rows occurred multiple times within the family Didemnidae, assuming that the ancestral didemnid had four rows of stigmata. In the present study, at least four distinct groups (*Trididemnum* 1–4) were recognized in the universal tree (Fig. 3) and two of them form a monophyletic clade with *Didemnum*. Kott [11] recognized four species groups in *Trididemnum*, i.e., *savignii* group, *miniatum* group, *cyclops* group, and *dispersum* group, but these four species groups do not correspond to the four groups *Trididemnum* 1–4 in Fig. 3. In Fig. 4, the didemnid species lacking an atrial siphon formed a single clade, but the bootstrap support for this clade was only 77% (ML). This may suggest the possibility that a lineage lacking an atrial siphon diverged from a lineage with an atrial siphon. A reduction in stigma rows may have occurred multiple times in both lineages with and without atrial siphons, generating *Trididemnum* 1 and 2 in Fig. 3 in the lineage with atrial siphons and *Trididemnum* 3 and 4 within *Didemnum* that always lack atrial siphons. While the polyphyletic group *Trididemnum* includes species with various modes of photosymbiosis, a broader phylogenetic analysis is needed to understand the phylogenetic relationships of each group in the family Didemnidae.

The genus *Didemnum* is the second largest ascidian genus, including over 240 species [5], and is characterized by a coiled vas deferens, lack of atrial siphon, and 1–2 testis lobes. The monophyly of *Didemnum* species, including *T. miniatum* and *T. nubilum*, was supported by high bootstrap values in both the universal and combined trees. However, these phylogenetic trees probably cover only a small part of the diversity within this genus, considering the large number of *Didemnum* species present. In the universal tree (Fig. 3), this *Didemnum* clade was in a polytomic relationship with the *Polysyncraton, Diplosoma*, and the two clades of *Lissoclinum*. These trees suggest that the genus *Lissoclinum* may be divided into two or more subgroups.

In *Lissoclinum* and *Diplosoma*, the vas deferens is uncoiled. Tunic spicules are always found in *Lissoclinum* but never in *Diplosoma*. The monophyly of these species with uncoiled vas deferens was supported in the complete 18S rRNA tree [9] and the partial *COI* tree [32] with high bootstrap values, and in the partial *COI* (Yokobori region) tree with low bootstrap values [44] but not supported in the present universal and combined trees (Figs. 3 and 4). The genus *Diplosoma* is characterized by the absence of tunic spicules, four rows of stigmata, and an uncoiled vas deferens. The monophyly of this genus is well supported in the present trees (Figs. 3 and 4) as well as in the trees of preceding studies [8, 32].

This study suggests some points of agreement and disagreement between morphological traits and molecular phylogeny. However, considering the vast diversity and number of species in Didemnidae, further analysis using more species and genetic markers is needed to verify these possibilities.

### Phylogeny of didemnid genera and photosymbiosis traits

To date, obligate symbiosis with cyanobacteria is known in four of the nine didemnid genera: *Didemnum*, *Diplosoma*, *Lissoclinum*, and *Trididemnum*. Among the four genera encompassing over 430 species in total [5], photosymbiotic species comprise only approximately 30 species, all of which inhabit tropical and subtropical regions. Preceding studies on didemnid phylogeny [7–9, 32, 44] and the present analyses did not support the monophyly of photosymbiotic species but almost supported the monophyly of each of *Didemnum* and *Diplosoma*. Of the approximately 250 *Didemnum* and 40 *Diplosoma* species, obligate photosymbiosis has been observed in fewer than ten species in each genus; thus, most congeners of the obligatory photosymbiotic species in each genus are non-symbiotic species. While *Lissoclinum* is divided into two clades in the universal tree (Fig. 3), each clade includes both symbiotic and non-symbiotic species. Therefore, it is unlikely that a single common ancestor of didemnids established an obligatory symbiotic relationship with cyanobacteria and subsequently lost the symbiotic association in most species within each genus. It is more reasonable to assume that phototrophic symbiosis arose within each lineage after the respective genus appeared, and in some cases, it occurred multiple times within a genus. To clarify the process of establishing photosymbiosis, it is necessary to investigate the phylogenetic relationships between symbiotic and non-symbiotic species by incorporating more species within each lineage to which photosymbiotic species belong.

**Fig. 5:**
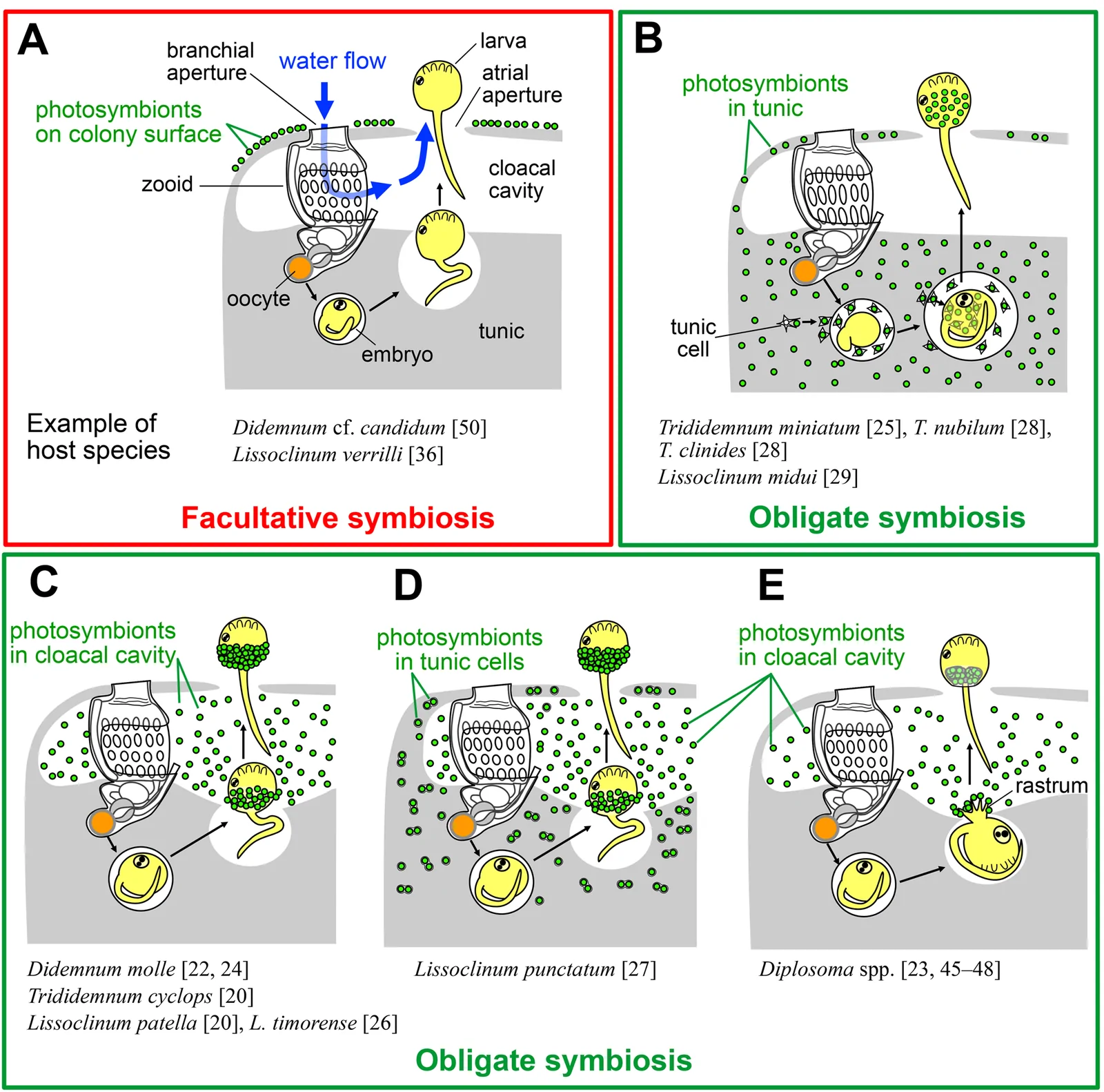
Schematic drawing of the modes of facultative (**A**) and obligate (**B–E**) photosymbiosis in didemnid colonies with remarks on the location and vertical transmission of photosymbionts. (**A**) Cyanobacterial cells on the colony surface are not transferred to ascidian larvae. (**B**) Photosymbionts (*Prochloron, Synechocystis*, or filamentous cyanobacteria) in the tunic are captured and carried by tunic cells to the presumptive adult tunic of the larvae. (**C**) *Prochloron* cells in the peribranchial and cloacal cavities are directly attached to the larval trunk. (**D**) *Prochloron* cells contained in the tunic cells are not transferred to the larva in *Lissoclinum punctatum*. (**E**) *Prochloron* cells in the peribranchial and cloacal cavities are collected with the rastrum of the pre-hatching larva in *Diplosoma* species

Several modes of photosymbiosis are known in didemnid-cyanobacterial symbiosis, characterized by cyanobacterial species, locations of symbionts within the host, and mode of vertical transmission (Table 1, Fig. 5). As shown by the symbols in Fig. 4, photosymbiosis with the same character states is found across multiple clades and genera, suggesting that a similar mode of photosymbiosis has evolved multiple times within Didemnidae. Vertical transmission has not been reported in species harboring cyanobacteria on the tunic surface, and symbiosis is always facultative in these species (Fig. 5A). In contrast, symbiosis is always obligate in species exhibiting vertical transmission of photosymbionts, and the modes of vertical transmission are similar in association with the distribution pattern of photosymbionts within the host colony, except for *Diplosoma* [3]. In species harboring cyanobacteria in the tunic, tunic cells transfer algal cells to embryos brooded in the tunic (Fig. 5B), and this mode is found in three distinct groups of *Trididemnum* [25, 28] and *Lissoclinum* [29] (red and/or green squares in Fig. 4). In species harboring *Prochloron* in the peribranchial and cloacal cavities, symbiont cells are directly attached to the larval trunk (Fig. 5C) in *Trididemnum* 2 [20], *Didemnum* [22, 24], and *Lissoclinum* [20, 26] (green circles in Fig. 4). In *L. punctatum* harboring *Prochloron* cells in both the tunic and cloacal cavities, *Prochloron* cells in the tunic are always located within the tunic cells, that is, tunic phycocytes [30]. However, these intracellular *Prochloron* cells are not involved in vertical transmission, whereas the *Prochloron* cells in the cavities are attached to the larval trunk [27] (Fig. 5D). Exceptionally, *Diplosoma* species exhibit a unique mode of vertical transmission (Fig. 5E). The pre-hatching larvae possess a unique organ (rastrum) for collecting *Prochloron* cells in the common cloacal cavity of their maternal colony [19, 21, 23] (green circles with black outline in Fig. 4). Kott [20] inferred that symbiosis involving the vertical transmission of photosymbionts was established only once in this genus, after which the extant photosymbiotic species were diversified. While additional photosymbiotic *Diplosoma* species have been described in the 2000s, the pre-hatching larvae of these species have also been found to possess a rastrum in all cases examined to date [33, 45–47]. Although the phylogenetic tree in this study does not contradict Kott’s view, it would be desirable to include more non-symbiotic species in future studies.

### Evolutionary history of photosymbiosis in didemnid ascidians

The molecular phylogenetic trees based on partial *COI* sequences did not support the monophyly of each photosymbiotic trait (cyanobacterial species, symbiont locations, and vertical transmission). Therefore, similar modes of photosymbiosis may have arisen in distinct didemnid lineages. Except for *Diplosoma,* which has a genus-specific mode of vertical transmission of photosymbionts, the modes of vertical transmission may have evolved convergently under a similar reproductive mode in didemnids, where embryos are brooded in the common tunic, larvae hatch into the common cloacal cavity, and swim out through the common atrial aperture.

Cyanobacterial cells occasionally colonize the colony surface, and this facultative association has been reported not only in didemnid ascidians but also in some non-didemnid ascidians [48–51]. In obligate symbiotic didemnids so far known, photosymbionts are always distributed in tunics or cavities, and these symbionts are transferred to the embryos or larvae of the host ascidians. Therefore, the establishment of obligate symbiosis likely requires two conditions: 1) habitat change for symbiotic algae from the colony surface to tunics or cavities and 2) the establishment of vertical transmission of symbionts. Molecular phylogenetic analyses suggest that these processes have occurred in several lineages within the family Didemnidae.

As discussed above, the didemnid lineage has likely had multiple opportunities to establish obligate symbiosis. Why is obligate symbiosis only found in didemnids among diverse chordate taxa? If vertical transmission of photosymbionts is a requirement for the establishment of obligate symbiosis, then the colony structure and sexual reproduction mode of didemnids may have been more conducive to the evolution of vertical transmission than those of other chordates. In didemnid photosymbiosis, symbionts are transferred to embryos or larvae brooded in the host colony. Since adaptive alterations in the sexual reproduction process are necessary to realize vertical transmission, the mode of reproduction is supposed to be an important factor in the establishment of obligate symbiosis. However, because only a small proportion of didemnid species establish obligate symbiosis with cyanobacteria, this exclusive photosymbiosis cannot be explained solely by the characteristics of the didemnid colonies. Therefore, there may be unknown conditions for obligate symbiosis, which may be the key to understanding the evolutionary processes underlying photosymbiosis.

## Electronic supplementary material

Below is the link to the electronic supplementary material.

Supplementary Material 1

## Data Availability

All data generated or analyzed during this study are included in this published article and its supplementary information files. Sequences are deposited in the DDBJ/EMBL/GenBank databases.
